# Recurrent Abortion and the Involvement of Killer-Cell Immunoglobulin-like Receptor (KIR) Genes, Activated T Cells, NK Abnormalities, and Cytokine Profiles

**DOI:** 10.3390/jcm12041355

**Published:** 2023-02-08

**Authors:** Mihaela Andreescu, Francesca Frîncu, Mihaela Plotogea, Claudia Mehedințu

**Affiliations:** 1 Department of Clinical Sciences, Hematology, Faculty of Medicine, Titu Maiorescu University of Bucharest, 040051 Bucharest, Romania; 2 Department of Hematology, Colentina Clinical Hospital, 020125 Bucharest, Romania; 3 Department of Obstetrics and Gynecology, Filantropia Clinical Hospital, 01171 Bucharest, Romania; 4 Department of Obstetrics and Gynecology, Carol Davila University of Medicine and Pharmacy, 020021 Bucharest, Romania; 5 Department of Obstetrics and Gynecology, Nicolae Malaxa Clinical Hospital, 022441 Bucharest, Romania

**Keywords:** pregnancy, immune tolerance, treg, genetics, intra-uterine, feto-maternal

## Abstract

Immune tolerance at the feto-maternal interface is crucial for the growth of the semi-allograft fetus in the womb. The outcome of pregnancy is dependent on a fine balance between various immunological forces. For a long time, the potential role of the immune system in pregnancy disorders has remained enigmatic. Current evidence has revealed that natural killer (NK) cells are the predominant immune cell population in the uterine decidua. NK cells cooperate with T-cells to provide an optimal microenvironment for the growth of the developing fetus by producing cytokines, chemokines, and angiogenic factors. These factors support trophoblast migration and angiogenesis which regulates the process of placentation. NK cells differentiate between “self” and “non-self” through their surface receptors known as killer-cell immunoglobulin-like receptors (KIRs). They induce immune tolerance through communication via their KIR and fetal human leucocyte antigens (HLA). KIRs are surface receptors of NKs that comprise both activating and inhibiting receptors. Due to the wide diversity manifested by its genes, the KIR repertoire is different in each individual. Significant evidence has implicated KIRs in recurrent spontaneous abortion (RSA); however, maternal KIR gene diversity in RSA is still unclear. Research has shown that immunological aberrancies including activating KIRs, NK abnormalities, and T cell downregulation are risk factors for RSA. In this review, we discuss relevant data from experimental studies on NK cell abnormalities, KIR, and T-cells in the incidence of recurrent spontaneous abortion.

## 1. Introduction

Pregnancy is a complex physiological event that involves extensive maternal-fetal dialogue at various levels. The successful outcome of pregnancy is dependent on fetal immune tolerance [1], hormonal balance [2], genetic predisposition [3], and environmental factors [4]. Throughout gestation, unprecedented intra-uterine events such as angiogenesis, endocrine regulation, and maternal immune system adaptations keep unfolding to ensure fetal growth and development [5]. The role of reproductive immunology in pregnancy and its disorders has always fascinated researchers; however, the role of immunology in feto-maternal settings remains poorly understood. Generally, the human immune system works based on discrimination between self and non-self [6]. Upon presentation of non-self antigens, the classical immune response is initiated which often culminates in the destruction of foreign particles. However, during pregnancy, such a hostile reaction is not exhibited by the maternal immune system [7]. The developing fetus is recognized as a semi-allograft due to the presence of paternal genes. To ensure fetal growth and avoid rejection, the maternal immune system undergoes various modifications by recognizing the fetus as a ‘temporary self’ [8]. However, alterations in protective expressions of the immune system result in adverse outcomes for pregnancy including implantation failure, preterm birth, recurrent spontaneous abortion (RSA), and preeclampsia [9]. In recent years, there has been an increased focus on understanding the immunological perspectives on pregnancy complications.

Spontaneous abortion is predominating disorder of pregnancy, with 10–15% of pregnancies culminating in early miscarriage. Although most of the abortion incidences are non-recurrent, a subset of 1% is recurrent in nature [10]. Several pathogeneses have been identified for RSA including genetic predisposition, environmental toxicities, hormonal imbalances, and infections. Chromosomal abnormalities are considered the most common contributor to spontaneous abortion, with 50% of spontaneous abortions being related to some genetic abnormality of the embryo [11]. Other major risk factors for RSA include the number of pregnancies, age at primary birth, and age at the last pregnancy [12]. The potential role immune system in pregnancy disorders has remained enigmatic for a long time; however, recent evidence has uncovered etiologies of immunological nature. A key concept to emerge in this regard is the disturbance in immune tolerance which refers to carrying the semi-allograft fetus to full term without rejection by the maternal immune system [13]. This is achieved by a tightly regulated cross-talk between maternal immune cells such as NK cells and T-cells and trophoblasts [14]. During pregnancy, the uterine decidua is populated with natural killer (NK) cells that constitute about a total of 30% of the total cellular content of decidual stroma. NK cells differentiate between “self” and “non-self” through their surface receptors known as killer-cell immunoglobulin-like receptors (KIRs). The successful outcome of pregnancy also depends on a proper inflammatory response [15], as adequately described in the following sections. The current review is aimed at presenting the current evidence on the potential role of KIR abnormalities in the predisposition of recurrent spontaneous abortion.

## 2. The Role of Inflammation in Pregnancy

Inflammation has emerged as a significant influencer in determining the outcome of pregnancy [16]. A significant body of evidence has demonstrated that T-helper (Th-17) produces excessive pro-inflammatory cytokines that can lead to decreased survival of the fetus [17,18]. The complexity of the immunological response in pregnancy is evident from the fact that both inflammatory and anti-inflammatory processes prevail during pregnancy and the outcome of pregnancy is based on the interplay of these processes. Significant evidence has revealed a limited inflammatory state during implantation, an anti-inflammatory state during mid-pregnancy, and renewed inflammation during parturition [19]. The need for inflammation during implantation and early trimester can be explained because blastocytes invade the uterine endothelium for implantation, and during the process, they damage the endometrium. To ensure post-damage repair, the inflammatory process is initiated for the effective repairment of epithelium. The need for inflammation during successful implantation is supported by Zhou et al. who reported that local injury to the endometrium improves the success rate of implantation [20]. The second trimester of pregnancy exhibits immune tolerance as the maternal-fetal relationship is symbiotic at this phase to encourage rapid fetal development. Therefore, anti-inflammation is the predominant feature at this stage. During the last phase of pregnancy, the inflammation process is again initiated to promote contraction and reject the placenta [21]. The sea-saw between pro and anti-inflammatory processes can influence the risk of infectious diseases as well. Pregnant women are more prone to malaria infection during the early part of their pregnancy, however, the risk decreases during the second half of gestation [22]. Adverse pregnancy outcomes are the consequence of one or more factors involving immunological impairment.

## 3. Natural Killer (NK) Cells and Their Role in Pregnancy

NK cells were initially characterized as innate lymphocyte subsets that mediate anti-tumor and anti-infected cells. Their discovery is a result of the unintended consequences of experiments focused on determining T cell-mediated cytotoxicity when cytotoxic lymphocytes with anti-tumor properties were uncovered [23]. Later, these cells were named natural killer cells. Unlike cytotoxic T cells, NK cells do not require pre-exposure to antigen or MHC restriction [24]. Recently, significant evidence has emerged on NK cell biology that elucidates the role of NK cells in pregnancy and RSA. The functionality of NK cells is regulated by the critical balance of activating and inhibiting signals that impart not only direct cytotoxic properties but also protective effects via cytokine production. The NK cells are classified into three distinct groups: the killer immunoglobulin-like receptors family, the C-type lectin family (CD94/NKGs), and the immunoglobulin-like transcripts (ILTs or LIRs). All receptor families have both activating and inhibiting members that recognize target cells and trigger NK cell cytotoxicity. The NK cell receptor repertoire is different in each individual. 

The number of NK cells during the pre-ovulatory stage is minimum; however, the NK cells start to increase during the secretory phase of the menstrual cycle as the progesterone levels increase. At the end of the menstrual cycle, the number of NK cells diminishes [25]. If the pregnancy ensues, NK cells are dominated type of cells in the decidual cell population where they consist of 70% of all mononuclear cells. Their number decreases with the gestation period and is reduced to a minimum at the end of the term. During placentation, NK cells express their complete activating receptor repertoire including NKp44 which is often reported after the activation of NK cells [26]. Some studies have reported that low levels of NKp44 can lead to reproductive failure [27]. However, a key point to understand is that uterine NK cells are different from peripheral blood cells. Uterine cells are mostly CD56^bright^CD16− whereas blood NK cells are CD56^dim^CD16+ [28]. They are believed to directly communicate with invading trophoblasts; therefore, they play a vital role in establishing normal pregnancy. The immunoregulation of the maternal immune response by NK cells is complex as they exhibit cytotoxic activity to control trophoblast invasion while local immunomodulation is via TH2- and TH3-type cytokines. However, they can express classical NK cytotoxicity and alloimmune reactions resulting in the identification of the fetus as “non-self” [29].

### 3.1. NK Cell Cytotoxicity and Recurrent Spontaneous Abortion

The role of NK cell cytotoxicity in adverse pregnancy outcomes is well established. Hadinedoushan et al. evaluated whether the NK cell cytotoxicity is altered in RSA patients or not. Their findings showed a higher NK cytotoxicity in 21 patients with RSA within 24 h after their abortion. The investigators concluded that NK cytotoxicity and increased IL-2 presence could be risk factors for recurrent abortion [30]. Similar observations were made by Yamada et al. who reported that preconception NK abnormalities were a significant cause of recurrent abortions in females [31]. Their findings were based on 113 RSA women who showed high values of pre-conceptional NK cell activity and later experience spontaneous abortion. A study by Karami et al. found that NK cytotoxicity was significantly higher in RSA subjects compared to healthy control participants (*p* < 0.001) [32]. Shakhar et al. found that the levels of NK cells and their activity varies with the number of recurrent abortions. Their results revealed that primary aborters had the highest concentration and activity of NK cells followed by secondary aborters whereas least levels of NK cells were found in control patients [33]. Yamada et al. correlated the higher NK cell activity with recurrent abortions. Their results found that higher NK cell activity at 6–7 gestation weeks culminated in abortion in RSA women [34]. Immunotherapy has been suggested by Perricone et al. to reduce the high levels of NK cells in RSA patients [35]. Fukui et al. found that the expression of CD56(bright)/a2V-ATPase+ cells was upregulated in RSA women and NKp46, NKp44, NKp30, and a2V-ATPase on CD56(bright) NK cells was also increased compared to CD56 (dim) NK cells [36]. Thum et al. investigated the predictor of successful IVF outcomes. Their finding showed that serum tumor necrotic factor (TNF)-alpha and interferon (IFN)-gamma levels had no relation with recurrent abortion, however, high levels of TNF-alpha and IFN-gamma are associated with elevated levels of activated NK cells which could be a risk factor subsequent abortion [37]. The cytotoxicity of NK cells is regulated by activating and inhibitory receptors and they can be determinants of NK cell activation. CD94 is an inhibitory receptor and CD 69 is activating receptor for NK cells. Ntrivalas et al. reported that RSA patients have significantly decreased CD 94 levels whereas elevated levels of CD 69 in their peripheral blood [38].

### 3.2. NK Cells and Their Cytokines in RSA

To ensure immune tolerance during gestation, a balance of inhibiting and activating NK receptors and cytokines production play fundamental roles. Two types of NK cells including NK1 and NK2 produce different types of cytokine population at the feto-meternal interface. NK1 cell types produce IFN-γ and Tumour necrosis factor-α (TNF-α) whereas NK2 cells release IL-4, IL-5, IL-10, and IL-13 [39]. IL-10 has been regarded as a key cytokine in the success of pregnancy whereas IFN-γ and TNF-α promote angiogenesis and trophoblast cell growth. Abnormal levels of cytokines have been shown to influence negative outcomes in pregnancy [40]. Dong et al. reported that Th1/Th2 and dNK1/dNK2 levels are higher in women with recurrent miscarriage history compared to control [41]. Fukui et al. found a lower concentration of IFN-γ and TNF-α-producing uterine NK cell recurrent spontaneous miscarriage [27]. However, Comba et al. reported higher levels of IFN-γ in RSA females along with IL-12 and IL-18 whereas leukemia inhibitory factor (LIF), and migration inhibitory factor (MIF) levels were lower in their study [42]. Recently, NK22 has been identified in uterine NK cells and it produces IL-22. NK-22 is believed to promote the regeneration of endometrium and regulate trophoblast invasion. According to Wang et al. investigation, lower levels of IL-22 might be involved in recurrent spontaneous abortions [43].

## 4. KIR and HLA Interaction

KIR genes manifest a wide diversity as it encodes for inhibitory and activating genes that are expressed not only on NK cells but also on CD4, CD8, and γδ T cells [44]. The allelic diversity makes it difficult to have similar KIR genotypes among individuals. The KIR receptors are the most studied NKRs. They are glycoproteins in nature. KIRs are further divided into two subfamilies based on Ig-like domains: the KIR2D (2 domains) and KIR3D (3 domains). Activating KIRs have short (S) cytoplasmic tails such as 2DS, whereas inhibitory KIRs have long (L) cytoplasmic tails such as 2DL and 3 DL. The KIRs interact with the human leukocyte antigens (HLAs)—the complementary ligands that help identify the self from the non-self [45]. HLA-Cs are members of HLA class I molecules that are expressed on trophoblast cells. The interaction between KIR and HLA class I molecules regulates the activity of NK cells and also imparts protective effects in pregnancy. The mechanism of activation of NK cells involves the suppression of inhibitory receptors and eliciting behavior of activating KIR that helps in the identification of non-self antigens. Most of the family members of KIR bind to HLA-C allotypes. 

The interactions between KIR and HLA on trophoblast result in immune tolerance [46]. However, both KIR and HLA genes are polymorphic which means that the outcome is based on a different type of KIR molecule binding with different types of HLA molecules. KIRs belong to a diverse family of activating and inhibiting clusters of receptors including six activating and eight inhibitory receptors which are located on chromosome 19q13.4 [47]. Although some KIR genes such as KIR2DL4 are present in every individual, the haplotypic diversity of the KIR locus varies greatly among individuals and ethnicities [48]. KIR2DL4 has been shown to recognize the HLA-G and can pair with the FcγR adaptor to trigger NK cell activation. The halophytes of KIR are grouped into A and B based on the gene content [49]. A-type halophyte has seven genes including KIR2DL4 and KIR2DS4 which are activating in nature but mostly unfunctional. The KIR2DS4 presence varies widely among the population with almost 80% of Caucasians having mutations that prevent its functionality. KIR B halophytes have a higher number of genes which varies between four to fifteen [50]. As already discussed, due to the variations between KIR and HLA genes, each pregnancy is unique in terms of the KIR and HLA-C combination. The receptor-ligand interaction mediates human immunity and can influence the disease pathogenesis of recurrent abortion.

### Clinical Evidence on KIR and RSA

The role of KIR in recurrent is not well-established and the studies published in this regard are often conflicting (Table 1). A meta-analysis by Akbari et al. demonstrated the detailed relationship between KIR and recurrent abortion [13]. Their investigation revealed a statistically significant relationship for only three types of KIR genes including 3DL1, 2DS2, and 2DS3, exhibiting protective, risk, and risk effects impacts, respectively. After multiple test correction adjustments, the findings did not remain significant. Their meta-analysis reported significant bias in many studies. A previous study by Akbari et al. reported that KIR2DS1 along with paternal HLA-C2 can be a risk factor for RSA [51]. These findings were supported by Wang et al. who found that activating KIR genes such as KIR2DS1 is a risk factor for RSA in Chinese women. Their findings showed that there is an increase in activating KIR genes and decreased HLA-C alleles in RSA patients [52]. This evidence can explain the immunological pathogenesis of RSA that decreasing ligands for inhibitory KIRs can result in higher NK cell activation in RSA patients. Contradicting results were reported by Hiby et al. among the Caucasian population. They found that activating KIR (KIR2DS1) for HLA-C2 was lower in RSA women [53]. 

Another study among the Brazilian population by Witt et el. reported that KIR polymorphism is not linked with recurrent abortion. KIR repertoire was similar in terms of inhibitory receptors and activating receptors in the control and RSA group [54]. Wang et al. reported that the expression of KIR2DL1/S1 on NK cells was significantly lower in RM women compared to control group [55]. Yamada et al. were able to establish a link between KIR repertoire and RSA. They recruited 20 RSA women and analyzed markers of NK cells such as CD94, CD158, and CD158a through flow cytometry in peripheral blood. Their results indicated that a decrease in CD158a expression was demonstrated in RSA women compared to the control [56]. However, the small sample size was the limitation of their study An interesting finding was reported by Varla-Leftherioti et al. who found that RSA women did not have the full KIR repertoire. Their results showed that RSA women did not have inhibitory receptors such as KIR 2DL1, 2DL2, and 2DL3 to attach with the HLA-Cw ligand to inhibit NK [57]. Dambaeva et al. reported that among KIR2DS1-positive women, the co-expression of HLA-C2 is associated with RSA [58]. Vargas et al. found a risk association of activating KIR genes for RSA in the Brazilian Caucasian population. Their findings showed that several activating KIR genes are prevalent in RSA women compared to the control group [59]. Similar findings were reported by Faridi et al. who found that activating KIR were more prevalent in RSA patients compared to their control counterparts [60]. 

A study by Nowak et al. suggested that an excess of inhibitory KIRs was associated with miscarriages whereas a balance between activating and inhibitory KIRs is seen in healthy subjects [61]. In a later study, Nowak et al. identified that heterozygote HLA-C in KIR AA women impart a protective effect and ensure normal pregnancy [62]. Khosravifar et al. were able to establish a relationship between RSA and maternal HLA-C2. They investigated maternal KIR and paternal HLA-C and their association with RSA in the Iranian population [63]. Ozturk et al. established the protective role of the KIR AA genotype. Their findings showed that activating KIR genes were significantly higher in the RSA group compared to the control population [64]. A study by Alecsandru et al. revealed that the KIR AA haplotype is a risk factor for the success of double embryo transformation (DET) [65]. Nowak et al. investigated the role of KIR2DL4, LILRB1, and HLA-G in 227 couples and found a protective effect of women’s heterozygosity in −716 HLA-G and LILRB1 in RSA patients. They also revealed interesting findings related to male partners of RSA women who contained more 9A/10A genotypes of KIR2DL4 gene carriers [66]. Dambaeva et al. found that activating gene KIR2DS1 is not a risk factor alone, however, in combination with HLA-C2 can become a risk factor for RSA [58]. Overall, conflicting clinical data is reported concerning KIR and RSA which makes it difficult to draw a conclusion.

## 5. T-Cells and Their Role in Pregnancy and RSA

In response to the semi-allogenic embryo, the CD4 + T cells differentiate into various T cells to mediate a response including helper cells and regulatory T cells. The combination of CD4 + Th1 produces various cytokines that are lethal for infected cells [67]. However, in pregnancy Th1 and Th2 cells show physiological changes with the Th2 type present in an abundant amount at the maternal-fetal interface which imparts protective effects. An overexpression of Th1 cytokines is implicated in RSA [68]. Th-17, a subset of CD4 + T cells, can also produce excessive pro-inflammatory cytokines that can lead to decreased survival of the fetus. A detailed role of Th-17 in RSA was provided by Wang et al. They reported that Th17-related factors, IL-17, IL-23, and retinoid orphan nuclear receptor (RORC) mRNA were significantly higher in peripheral blood and decidua of RSA women compared to control. They also found that IL-27, a key regulator of Th-17 and T-regulated cells was downregulated in RSA patients [69]. Another study found that IL-17 can contribute to late abortion instead of abortion during the early part of pregnancy [70]. Najafi et al. established a relationship between IL-17F gene polymorphism rs763780 as a risk factor for 85 RSA Iranian women [71]. However, contradicting results were demonstrated by Nakashima et al. who found that Th-17 remains the same throughout the pregnancy [72].

The role of regulatory T cells is widely acclaimed in imparting protective effects during pregnancy (Table 2). Wang et al. reported that Treg cells have been shown to possess a protective role against IL-17 in the mouse model. Also, the loss of Treg cells results in effector-T cells which results in inflammation and can cause implantation failure [73]. Mjösberg et al. found that the CD4dimCD25highFoxp3+ Treg population increases during the first trimester and decreases during the second trimester. Their findings revealed that progesterone plays are vital role in the maintenance of Treg levels [74]. Arruvito et al. uncovered the role of Treg cells in the menstrual cycle and its potential implication in RSA women. Their finding showed that the levels of CD4 + FOXP3+ Treg cells in the peripheral blood of unexplained RSA were significantly lower compared to healthy individuals. The lower number of Treg cells in the follicular phase of the menstrual cycle can be a predisposing factor for recurrent embryo loss [75]. Qian et al. reported that levels of IL-6, TNF-ɑ, and the Th17/Treg ratio were significantly higher in women with unexplained RSA compared to healthy subjects [76].

Sasaki et al. reported that the decidual population of CD4(+) CD25(bright) T cells was significantly lower compared to the control population. They also observed that a decrease in Treg cells is accompanied by an increase in Th17 cells. In line with their findings, Liu et al. reported that Treg cell frequency was lower in recurrent miscarriage subjects whereas an increased level of Th1 cells was also reported by the authors [77]. Zhu et al. reported that the ratio of Treg/Th17 and Foxp3/ROR-γt decreased in patients with URSA compared to healthy controls [78]. Winger and Reed have demonstrated that CD4(+) CD25(+) Foxp3(+) T cells can be a marker for assessing pregnancy failure risk. They recruited fifty-four females with a previous history of miscarriage. Their results showed that Treg levels with successful ongoing pregnancy were 0.72 vs. 0.37 in patients who miscarried in their first trimester [79]. Zenclussen et al. showed that only pregnancy-induced Treg cells have the potential to prevent embryo loss. They studied this observation by adoptive transfer of Treg cells from both normal pregnant and unpregnant mice; however, the protective effect was only demonstrated by pregnant mice. This shows that fetal exposure is required for Treg cells to pass on their immune tolerance [80].

**Table 2:** T-cells and their role in pregnancy and RSA.

Authors	Study Population	Methodology	Results	Outcomes
Nakashima et al., (2010) [72]	11 non-pregnant women; 30 first trimester pregnant women; 10, second-trimester pregnancy women; 12 third-trimester pregnancy women.	Flow cytometry	Most of the IL-17-producing cells were CD4+ T cells. Th17 cells number remained the same in pregnancy.	Th17 levels in peripheral blood lymphocytes remain the same throughout the pregnancy.
Wang et al., (2014) [73]	Mice (*n* = 40) were randomly assigned to one of four experimental groups.	Interferon (IFN)-γ, IL-4, TGF-β, and IL-10 in the decidual tissue was assessed by real-time RT-PCR and western blotting.	Normal pregnant CBA/J mice mated with BALB/c males which received transvaginal rIL-17 showed increased chances of miscarriage. Administration of rIL-17 resulted in a decrease in the production of TGF-β and IL-10 at both mRNA and protein levels (*p* < 0.05). Transfusion of Tregs before mating increased TGF-β and IL-10 mRNA and protein levels (*p* < 0.05).	IL-17 in the feto-maternal interface is a risk factor for RSA.
Mjösberg et al., (2009) [74]	Thirty-eight healthy pregnant women (pregnancy weeks 24–28).	Foxp3, CD127, and HLA-DR were determined by multicolor flow cytometry.	CD4dimCD25highFoxp3+ Treg population increases during the first trimester and decreases during the second trimester.	Progesterone plays are vital role in the maintenance of Treg levels.
Qian et al., (2018) [76]	Part 1: 155 subjects Part 2: 35 subjects with URSA and 40 subjects with normal pregnancy	Flow cytometry.	Levels of IL-6 and tumor necrosis factor-ɑ (TNF-ɑ) and the Th17/Treg ratio were significantly increased in RSA patients compared to the control.	levels of IL-6 and TNF-ɑ, and the Th17/Treg ratio at the maternal-fetal junction predict RSA.
Arruvito et al., (2007) [75]	Group 1: 75 women with a history of three or more consecutive RSA. Group II: 60 fertile women (who had at least one successful pregnancy and no previous abortions). Group III: 55 postmenopausal women. Group IV: comprised 52 men.	Flow cytometry	The levels of CD4+ FOXP3+ Treg cells in the peripheral blood of unexplained RSA were significantly lower compared to healthy individuals.	The lower number of Treg cells in the follicular phase of the menstrual cycle can be a predisposing factor for recurrent embryo loss
Liu et al., (2013) [77]	30 women (age: 32.5 ± 3.8) with RM at least three times.	The sperm antigen-specific T-cell response was assessed by flow cytometry.	Low frequency of sperm-specific Tregs and high frequency of T helper (Th)1 cell were detected in RM women as compared with women without RM	Downregulation of Treg is a risk factor for RSA.
Winger and Reed (2011) [79]	Fifty-four pregnant women with a history of immunologic infertility and/or pregnancy loss.	Flow cytometry for peripheral blood mononuclear cells T-regulatory-cell assay Th1:Th2 cytokine assay Antiphospholipid antibodies Antinuclear antibodies	Patients with successful ongoing pregnancies experienced a mean (CD4(+) CD25(+) Foxp3(+)) ‘Treg’ level of 0.72 ± 0.52%, while those that miscarried in the first trimester experienced a mean Treg level of 0.37 ± 0.29% (*p* = 0.005).	Decreased Treg levels are markers for assessing miscarriage risk.
Zenclussen et al., (2005) [80]	DBA/2J-mated mouse females (*n* = 28).	Flow Cytometry Real-Time Reverse Transcriptase (RT)-Polymerase Chain Reaction (PCR) Sodium Dodecyl Sulfate-Polyacrylamide Gel Electrophoresis and Western Blot Immunohistochemistry Mixed Lymphocyte Culture: Cytokine Secretion Assay	A higher frequency of interferon-γ-producing T cells specific for paternal antigens was seen in aborted mice compared to those from normal pregnancy (7.8% versus 2.7%, *p* < 0.05). Normal pregnant mice showed strongly elevated numbers of CD4 + CD25+ and interleukin-10+ Treg compared to aborted mice.	Pregnancy-induced Treg cells have the potential to prevent embryo loss.
Zhu et al., (2017) [78]	25 URSA patients (mean age of 29.08 + 2.90 years)	Flow Cytometry	The ratio of Treg/Th17 and Foxp3/ROR-γt decreased in patients with URSA compared to healthy controls. The serum levels of interleukin (IL)-6 and IL-17A were significantly higher, whereas IL-10 was lower in URSA patients compared with controls.	Treg and Th17 cells are implicated in RSA pathogenesis.

## 6. Conclusions

Although conflicting clinical data is available regarding immunological processes in recurrent spontaneous abortion, the role of NK cells and T cells is discussed significantly in immunopathology. The outcome of pregnancy is based on multiple immune factors including non-cytotoxicity of NK cells, cytokine population, and Treg cell involvement. Alterations in normal immunological response have been implicated in the loss of tolerance and subsequently miscarriage. The role of immune cells seems to be well-defined in pregnancy. Both NK cells and T cells provide a protective effect on developing fetuses in normal pregnancy. The role of KIR, a receptor of NK cells is still unclear in RSA. There is a lack of consensus among researchers in this regard. Some studies identify activating KIR while others implicate inhibiting KIR receptors in RSA. The current review found that critical immune mediators are involved in the pathogenesis of RSA, however, further studies are required to establish a clear role of KIRs, NK abnormalities, and T cells in recurrent abortion.

## Figures and Tables

**Table 1:** The role of KIR in the predisposition of RSA.

Authors	Study Population	Methodology	Results	Outcomes
Akbari et al., (2018) [51]	100 couples; females with a history of at least 3 abortions	Polymerase chain reaction with sequence-specific primers (PCR-SSP)	KIR2DS1 and paternal HLA-C2 were higher in RSA couples compared to the control. A significant relation was found for maternal C1C2 in combination with paternal C1 or C2.	Activating the KIR receptor in combination with HLA-C2 is a risk factor for RSA.
Wang et al., (2007) [52]	73 childless couples with a history of three or more abortions. 68 healthy control participants.	15 selected KIR genes and two groups of HLA-C alleles were identified by sequence-specific primers (PCR-SSP).	The KIR2DS1 gene was significantly higher in RSA subjects compared to the control. More than two activating genes were reported in the majority of participants. Couples with the maternal KIR2DS1 gene and with a decreased group 2 HLA-C allele for the homologous inhibitory receptor KIR2DL1 were abundant in the RSA group.	Maternal KIR2DS1 along with paternal 2HLA-C is a risk factor for RSA.
Hiby et al., (2008) [53]	Male (*n* = 67) Female (*n* = 95) with three or more spontaneous miscarriages.	HLA-C groups and 11 KIR genes were genotypes by using the PCR-sequence-specific primer method (SSP).	The frequency of the HLA-C2 group was increased in both parents in the RSA group (*p* = 0.018). The KIR gene frequencies were insignificant. Women had a high frequency of KIR AA haplotypes that lack activating KIR. In particular, the activating KIR for HLA-C2 groups (KIR2DS1) was significantly lower in these women.	HLA-C2 is a male risk factor for RSA.
Witt et al., (2004) [54]	51 Brazilian Caucasian women (18–45 years) History of three or more spontaneous miscarriages (mean 4, range 3–7).	Genotyping of KIR repertoire by SSP and SSCP.	Thirty-five percent of controls and 50% of patients had all three HLA-C inhibitory KIR (P ¼ 0.22) (KIR2DL1, KIR2DL2, KIR2DL3).	KIR polymorphism and RSA is not related.
Wang et al., (2014) [55]	30 RM women (aged 32.3 ± 7.22 years)	Immunofluorescence analysis for the expression of CD56, CD158a and CD158b.	CD56+ NK cells constituted 66.7 ± 16.9% of leukocytes in decidua of controls. A significant decline was seen in the frequency of CD56 + CD16− NK cell staining for KIR2DL1/S1 and KIR2DL2/S2/L3 throughout the first trimester in RM patients (*p* < 0.05).	RM is associated with a decline in the frequency of decidual NK cells expressing KIR specific for human leukocyte antigen (HLA)-C.
Yamada et al., (2004) [56]	20 RSA women 15 fertile controls	Perforin, CD94, CD161, CD158a, CD158b, and CD244 on CD3- CD56+ NK cells identification by flow cytometry.	A significant decrease in CD158a expression was demonstrated in RSA women compared to healthy subjects (*p* < 0.05). The expression of perforin, CD158b, or CD244 in RSA women did not differ from that in the controls.	A decrease in CD158a expression is seen in RSA.
Varla-Leftherioti et al., (2005) [57]	30 women with RSA history	DNA extracted from isolated decidual and trophoblastic cells was used for molecular detection of maternal inhKIRs (2DL1, 2DL2, 2DL3) and fetal HLA-Cw alleles, respectively.	60% of aborted subjects did not have the full repertoire of three inhKIRs. Limited epitope matching in RSA patients (less than three inhKIRs with specificity for fetal HLA-Cw alleles).	Lack of inhKIRs to interact with the HLA-Cw is a risk factor in recurrent abortion.
Dambaeva et al., (2016) [58]	139 Caucasian women with RSA.	The frequencies of KIR and HLA-C1 and HLA-C2 genes were evaluated.	45.3% participants were positive for KIR2DS1 and had exhibited higher frequency of its ligand, HLA-C2.	KIR2DS1-positive women with higher HLA-C2 expression have a higher incidence of RSA.
Vargas et al., (2009) [59]	68 south Brazilian Caucasian couple with a history of three or more miscarriages; 68 control fertile couples.	KIR genes were genotyped by PCR-Reverse SSO method.	Significantly higher activating KIR genes in the RSA group compared to the control.	Higher NK activation by activating receptors is a risk factor for RSA.
Faridi et al., (2009) [60]	205 RSA patients; 224 healthy controls.	Gene-specific PCR amplification (PCR-SSP) for KIR genotypes.	Activating KIRs was significantly higher in RSA patients compared to the control group.	Activating KIRs risk factor for RSA.
Nowak et al., (2009) [61]	149 couples (women: 32.44) History of three or more spontaneous abortions. 117 health controls; History of at least two healthy births	four multiplex PCR-SSP for KIR genotyping.	activating-to-inhibitory KIR gene ratios of 0.33 to 0.83 were seen in RSA patients.	Inhibitory KIRs as a risk factor for RSA.
Nowak et al., (2011) [62]	125 couples with RSA 117 control couples	four multiplex PCR-SSP for KIR genotyping.	In the group of KIR AA women with HLA-C C2C2 partners, the HLA-C C1C2 heterozygotes were present in the controls but not in the patients.	KIR AA women with HLA-C C2C2 partners and HLA-C heterozygous females improve pregnancy outcomes.
Khosravifar et al., (2011) [63]	92 couples and 8 women with RSA history. 100 healthy controls	HLA-C groups and 5 KIR genes (KIR2DS1, KIR2DS2, KIR2DL1, KIR2DL2, KIR2DL3) genotyping by using PCR-sequences-specific primer method (SSP).	HLA-C2 group is significantly higher in the RSA group compared to the control.	Maternal HLA-C2 is a risk factor for RSA.
Ozturk et al., (2012) [64]	40 women with RSA history 90 controls	Sequence-specific oligonucleotide probes analysis for 16 KIR genes.	The elevated number of activating KIR genes was significantly higher (*p* = 0.014) in women with recurrent miscarriages when compared with the control group.	Activating KIRs is a risk factor for RSA.
Alecsandru et al., (2014) [65]	291 women, with RSA.	KIR haplotype regions were defined by the presence of the following KIR genes: Cen-A/2DL3; Tel-A/3DL1 and 2DS4; Cen-B/2DL2 and 2DS2; as well as Tel-B/2DS1 and 3DS1.	Higher rates of miscarriages in subjects with the KIR AA haplotype (22.8%) followed by those with the KIR AB haplotype (16.7%) compared with mothers with the KIR BB haplotype (11.1%) were observed (*p* = 0.03).	KIR AA haplotype is a risk factor for the success of double embryo transformation (DET).
Dambaeva et al., (2016) [58]	139 Caucasian women with a history of two or more pregnancy losses.	Genomic DNA was extracted using QuickGene DNA.	The HLA-C1 and HLA-C2 group distribution were significantly different between women with or without KIR2DS1. Women positive for KIR2DS1 (45.3% of the study cohort) had an increased frequency of its ligand, HLA-C2.	Our results indicate that among KIR2DS1 pos women, the co-expression of HLA-C2 is associated with RSA

## Data Availability

Not applicable.
